# Toward Sustainability: Trade-Off Between Data Quality and Quantity in Crop Pest Recognition

**DOI:** 10.3389/fpls.2021.811241

**Published:** 2021-12-24

**Authors:** Yang Li, Xuewei Chao

**Affiliations:** ^1^College of Mechanical and Electrical Engineering, Shihezi University, Shihezi, China; ^2^School of Electrical and Information Engineering, Tianjin University, Tianjin, China

**Keywords:** feature engineering, classification, redundancy, low-shot, few-shot

## Abstract

The crop pest recognition based on the convolutional neural networks is meaningful and important for the development of intelligent plant protection. However, the current main implementation method is deep learning, which relies heavily on large amounts of data. As known, current big data-driven deep learning is a non-sustainable learning mode with the high cost of data collection, high cost of high-end hardware, and high consumption of power resources. Thus, toward sustainability, we should seriously consider the trade-off between data quality and quantity. In this study, we proposed an embedding range judgment (ERJ) method in the feature space and carried out many comparative experiments. The results showed that, in some recognition tasks, the selected good data with less quantity can reach the same performance with all training data. Furthermore, the limited good data can beat a lot of bad data, and their contrasts are remarkable. Overall, this study lays a foundation for data information analysis in smart agriculture, inspires the subsequent works in the related areas of pattern recognition, and calls for the community to pay more attention to the essential issue of data quality and quantity.

## Introduction

Crop pest identification using modern information technology is important to protect crop growth while optimizing the required human labor. This kind of intelligent plant protection is attracting much attention in the way of smart agriculture (Pathan et al., 2020). Based on the development of the Internet of Things and wireless sensor networks (Yang et al., 2020; Friha et al., 2021), image gathering is becoming easier in the agricultural field. Moreover, smart applications based on agricultural images have been widely emerging in many aspects of agriculture, such as plant disease identification (Nagasubramanian et al., 2019; Li and Chao, 2020; Li et al., 2020), crop pest recognition (Ayan et al., 2020; Liu and Wang, 2020; Mandal et al., 2021; Wang et al., 2021), fruits identification (Gao et al., 2020; Fu et al., 2021), yield forecasting (Schauberger et al., 2020; Shahhosseini et al., 2020; Jarlan et al., 2021), vision navigation (Kanagasingham et al., 2020; Rovira-Más et al., 2020; Emmi et al., 2021), and agricultural robot (Chen et al., 2020b; Guo et al., 2020; Wen et al., 2020; Zhang et al., 2020), etc. Although many remarkable achievements in the above typical aspects exist, the shortcomings of the current intelligent learning method are also revealed. Specifically, the current smart applications are mostly based on deep learning, which is a branch of machine learning and driven by big datasets.

But the collection and annotation of big datasets are not always feasible in agriculture, owing to the inherent long-tailed data distribution in nature (Chao and Zhang, 2021; Yang et al., 2021), it is very difficult to obtain enough data for some rare crop pests or diseases. In addition, this kind of learning approach based on massive data is resource-intensive and to some extent unsustainable. Some of the main reasons include the data cost, hardware cost, and power cost. Among them, the data cost refers to the human labor of obtaining rare data and the expert cost of data annotation. As known, the model to deal with big data is going deeper and deeper, while the deep model requires very expensive hardware with high performance to run, such as the high-end servers equipped with dozens of graphics processing unit (GPUs). Then, the power consumption of high-performance servers is staggering, which is unfavorable to achieving carbon peak and low carbon environmental protection (Chen et al., 2020a).

As mentioned, the current deep learning approach driven by big data and deep models are unsustainable to some extent. To move toward sustainable machine learning, the balance between data quality and quantity should be emphasized. Because the scale of the dataset can be reduced, the corresponding data acquisition and annotation cost will be low, the corresponding training model depth can be relatively shallow, and the required hardware cost and power consumption of neural network training will also be reduced.

The machine learning methods from limited data are also called few-shot learning, recently emerging in many interdisciplinary areas to focus on the pattern recognition from few data (Li and Yang, 2020, 2021; Li and Chao, 2021; Liang, 2021; Li et al., 2021). However, most of the related works are simply to reduce the number of samples and design the few-shot learning algorithms and to ignore the core analysis of data information. It should be noted that the way toward sustainable and smart agriculture should be established on limited data with high quality.

In this study, we proposed an embedding range judgment (ERJ) method based on the feature space to select samples with high information value and then adopted different depth networks to verify the validity of the proposed method on the relationship analysis between data quality and quantity in the crop pest identification. Extensive experiments show that the partial data can realize the same training effect of all data, the selection order of the proposed method is related to the representation performance of the used models, and the comparison between good data and bad data is clear and obvious. Thus, this study can provide important inspiration for future work to mine a limited amount of high-quality data to support sustainable pattern recognition in intelligent agriculture.

The rest of this study is arranged as follows. Section Materials and Methods describes the used dataset, overall framework, and algorithm flow of the proposed ERJ method. In section Results, the results of extensive comparison experiments are shown and analyzed. Discussions are carried out in section Discussions. Finally, section Conclusions concludes this study.

## Materials and Methods

### Crop Pest Dataset

**Figure 1:**
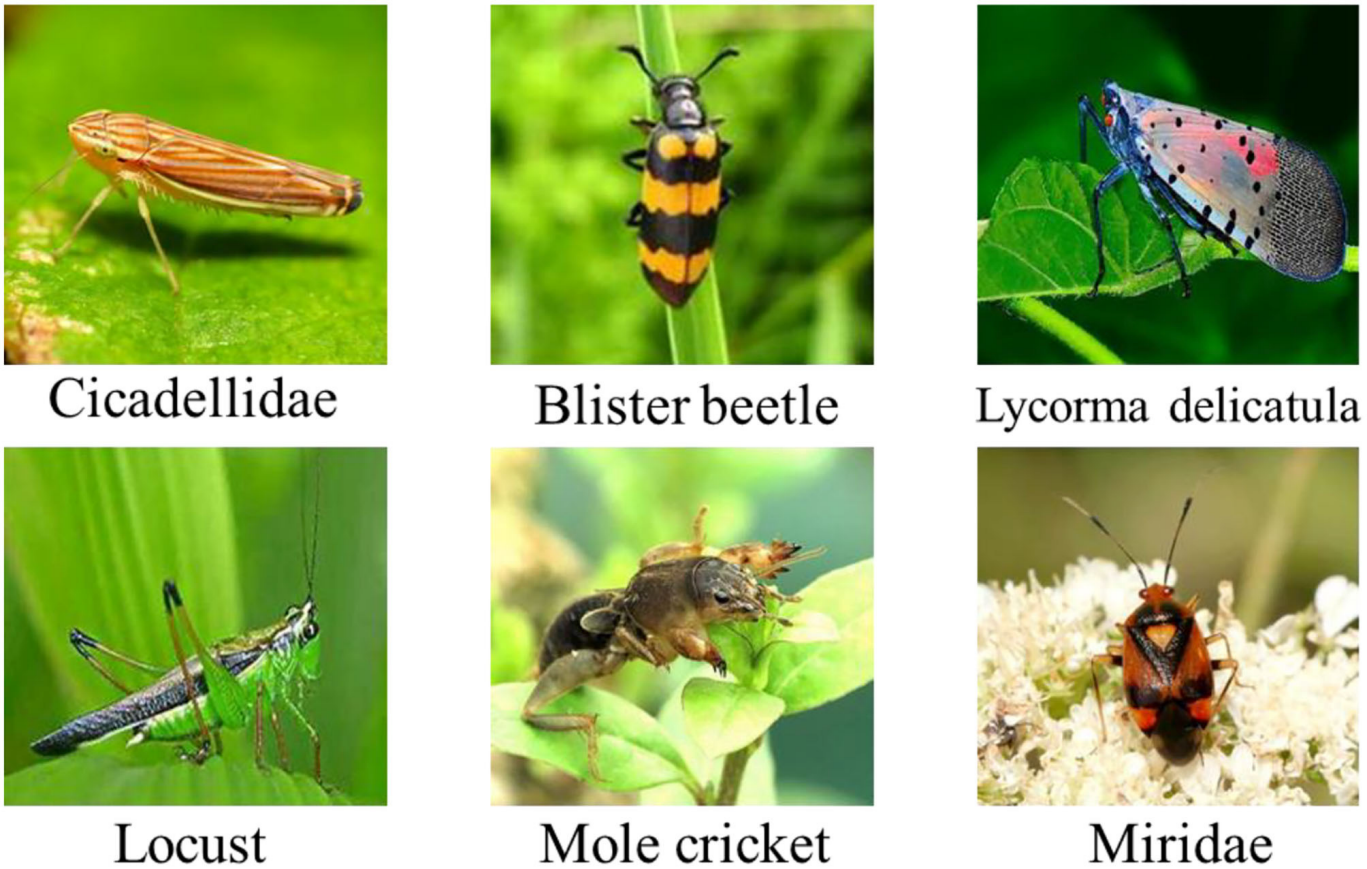
Some samples of the crop pest dataset.

Intelligent crop pest identification is critical to smart agriculture, which guides farmers to take suitable measures on time. In this study, we used a crop pest dataset with 6 categories, with 6,000 images in total. The dataset was class-balanced, including 1,000 images per category. The uniform size of red green blue (RGB) images was 224 × 224 × 3. Notably, the image backgrounds were in a natural environment, not in a simple lab environment. Some samples of the crop pest dataset are shown in Figure 1.

To test and compare the performance of convolution neural networks on the task of crop pest recognition, the adopted dataset was divided into a training set and test set at the ratio of 8:2, that is, all available training data had 800 images per category, and the rest of the 200 samples in each category were fixed for testing. To explore the trade-off between data quality and quantity, all the training data were just used to set a targeted level, guiding the selection of limited valuable samples.

### Overall Framework

**Figure 2:**
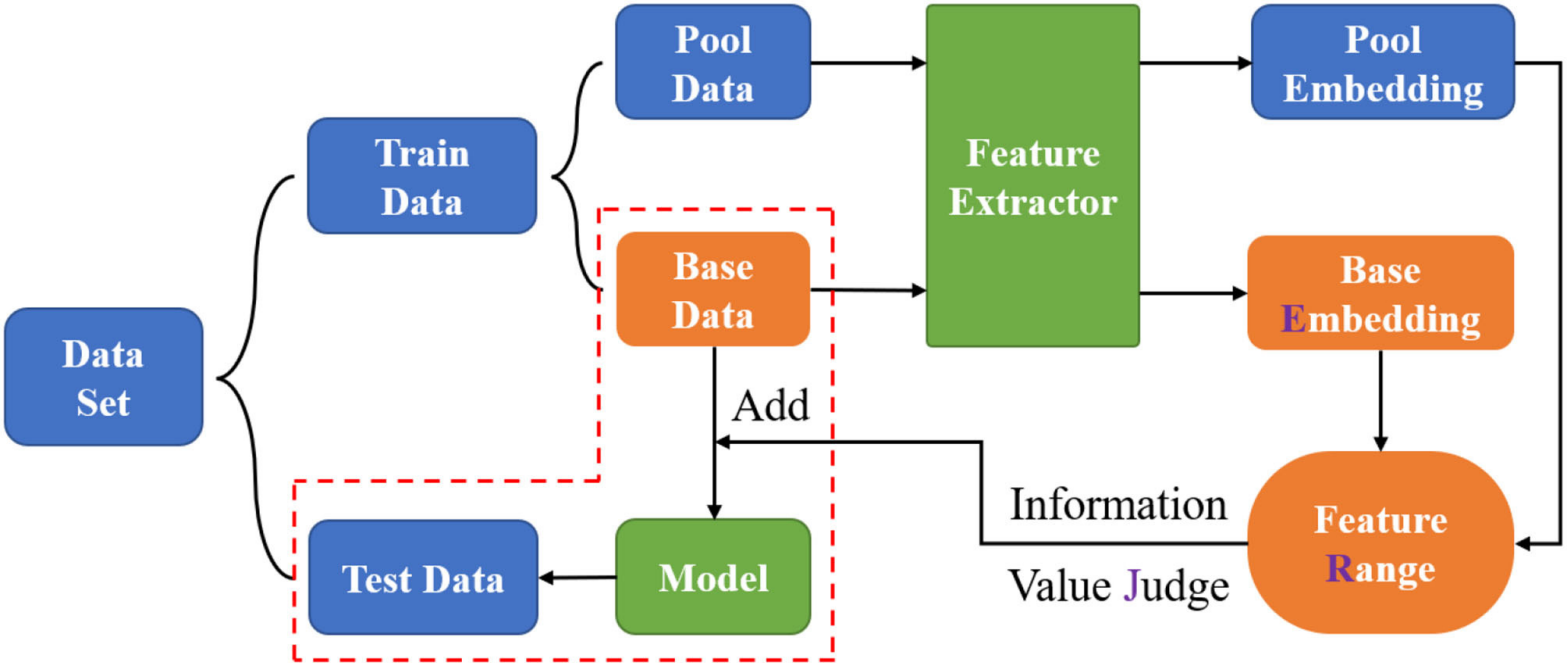
Overall framework.

The overall framework of this study is briefly shown in Figure 2, including the flow of the proposed ERJ analysis method and the process of exploring the trade-off between quality and quantity of image data.

In specific, the original dataset was first divided into two parts, namely, train data and test data at the ratio of 8:2, as mentioned earlier. Regarding the motivation for this study, all train data may not be necessary for the task, so the train data was divided into two parts, namely, pool data and base data, at a ratio of 9:1. In detail, the base data had 80 samples per category, while the pool data had 720 samples per category. Then, the feature extractor was first trained, also called fine-tuned, by the base data to extract the embeddings, which were high-dimensional vectors in the feature space. Next, a range of features corresponding to the existing base data can be formed. The pool data will be fed to the mentioned feature extractor to obtain the pool embeddings to compare with the feature range through every dimension. This process is called the information value judge in this study, which aims at selecting the samples with some dimensions outside the existing embedding range. Based on the data budget, the above operation can be iteratively carried out many times until enough data have been selected to achieve the targeted recognition performance. Finally, the selected good data are added into the base data to realize the trade-off between data quality and quantity, then the updated base data are used to train the neural network model.

### Algorithm

According to the above framework and operation descriptions, the proposed ERJ method should be clear to explore the data with high information value to the existing base data. Notably, this selection process is dynamic, the high information also varies from time to time, depending on the distribution of information already existing. In other words, for a fixed sample, when the existing base data change, the information value of the fixed sample will also change. The algorithm of the above descriptions is summarized in Table 1.

**Table 1:** Algorithm of the ERJ method.

**Input**: Base data S = {*S*_*B*1_, *S*_*B*2_, *S*_*B*3_···*S*_*B*6_}, where *S*_*B*_*i*__ = { *x*_1_, *x*_2_···, *x*_*N*_}. Pool data S = {*S*_*P*1_, *S*_*P*2_, *S*_*P*3_···*S*_*P*6_}, where *S*_*P*_*i*__ = { *x*_1_, *x*_2_···, *x*_*M*_}. As the initial split ratio of base data and pool data is 1:9, here *N* = 80, *M* = 720.
**Output**: Select data S = {*S*_*S*1_, *S*_*S*2_, *S*_*S*3_···*S*_*S*6_}, where *S*_*S*_*i*__ = { *x*_1_, *x*_2_···, *x*_*K*_}, *K* = 40.
**for model in {three depth models} do**
(1) Finetune parameters of model on the base dataset.
(2) Get the feature extractor from finetuned model.
(3) Feed the base data to the feature extractor to obtain the existing embedding range.
(4) Feed the pool data to the feature extractor to obtain the pool embeddings, and compare with the existing embedding range to judge the sample's information value. (5) If some samples have several dimensions outside the feature range, add it to the base data. Repeat this step until the data number reached 5% of whole data.
(6) Repeat the step 1–5 until the testing performance is satisfied or the data budget is full.
**end for**

## Results

The comparison experiments were carried out using different models to explore the validity of the proposed ERJ method to analyze the information value of data. The used experimental hardware is a sever with GPU of NVIDIA TITAN Xp (American Multinational Technology Company, Santa Clara, CA, USA), equipped with 12 GB memory, while the software environment is the Jupyter Notebook with libraries of TensorFlow (version 1.12.0), Numpy (version 1.19.2), Keras (version 2.2.4), and OpenCV (version 4.1).

The used three models with different depths are cut from the VGG-16 network, also called the shallow model, the middle model, and the deep model. The structure parameters of the three models are introduced as follows. The shallow model has 4 convolutional layers and 1 max-pooling layer, the middle model has 7 convolutional layers and 2 max-pooling layers, and the deep model has 13 convolutional layers and 4 max-pooling layers.

### High-Quality Data Selection Under Different Models

**Figure 3:**
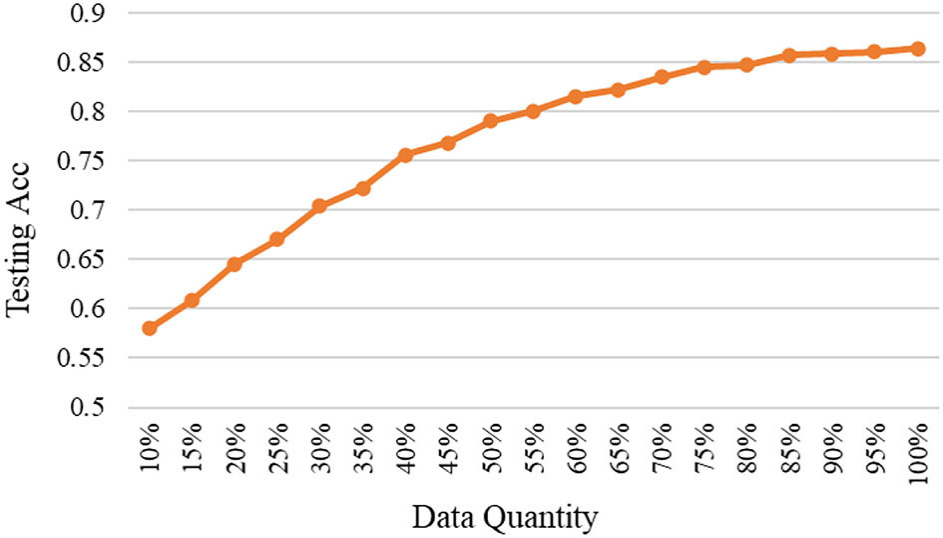
The relation between accuracy and data quantity under the shallow model.

**Figure 4:**
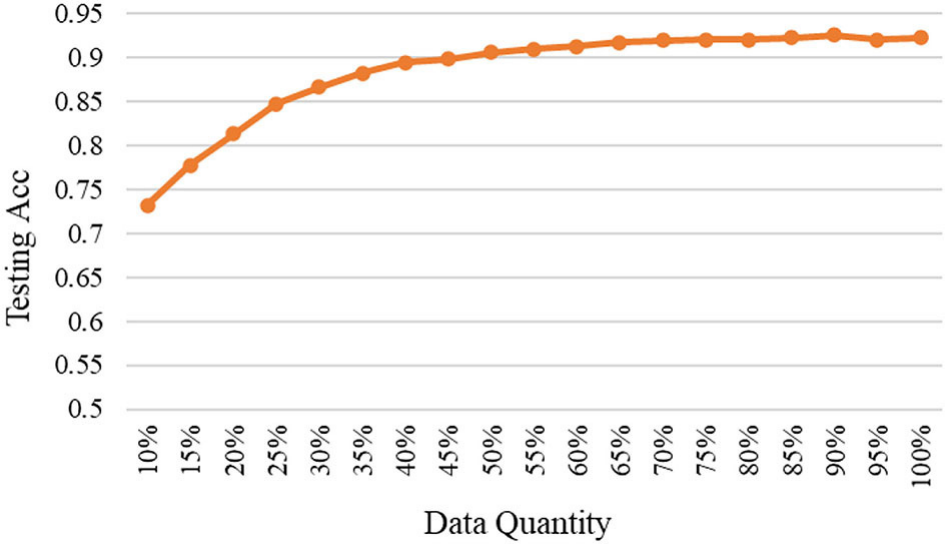
The relation between accuracy and data quantity under the middle model.

**Figure 5:**
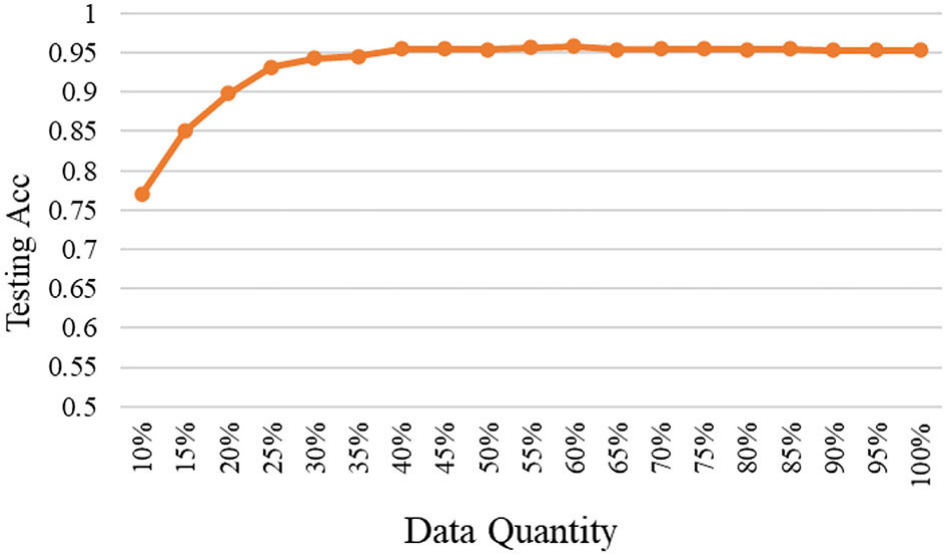
The relation between accuracy and data quantity under the deep model.

As mentioned, the selected data are added to the base data and used to train the model again, and then to test on the fixed testing data. The relationships between testing accuracy and the quantity of selected high-quality data under different models are shown in Figures 3–5. It should be emphasized that in this section, the proposed ERJ method is implemented by preferentially selecting samples with a relatively large number of dimensions whose features are out of the existing embedding range.

As shown, with the adopted shallow model, we selected high-quality data using the proposed ERJ method and found that there existing data redundancy in the dataset. It was shown that about 75% of selected data can reach the performance using all data.

As seen, with the adopted middle model, the effect is better than the shallow one, and the proposed ERJ method was used to select high-quality data. It was shown that about 60% of selected data can reach the performance using all data.

The deeper model generally has stronger representational ability, hence, the upper testing accuracies using all data are different under three models. Regarding the adopted deep model, using the proposed ERJ method to select high-quality data is significantly meaningful. It is shown that about 40% of selected data can reach the performance using all data.

In summary of this section, the proposed ERJ method is proved to be effective under different models, which means the ERJ method is robust. Using the ERJ method, it is feasible to achieve a small amount of high-quality data to achieve the same effect of all data, making the trade-off of data quality and quantity.

### Selection Strategy of the ERJ Method Under Different Models

The proposed ERJ method would like to select samples outside the existing feature range. But the data feature is high-dimensional, how many dimensions out of range are appropriate? Furthermore, how to set the data selection strategy and is it related to the model's representational performance? To explore the above questions, the comparison experiments were done by selecting a large number of dimensions and a small number of dimensions whose features are out of the existing embedding range.

**Figure 6:**
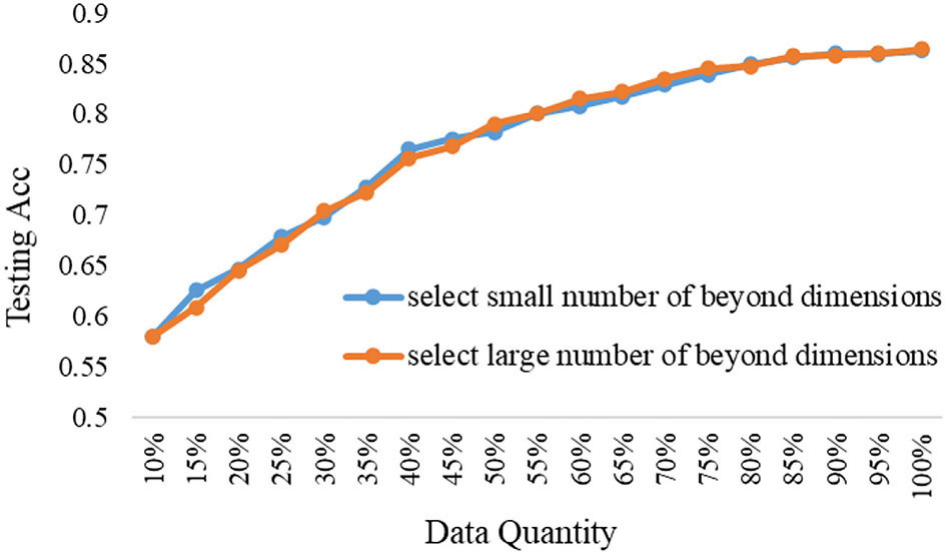
The data selection strategy of the ERJ method under the shallow model.

**Figure 7:**
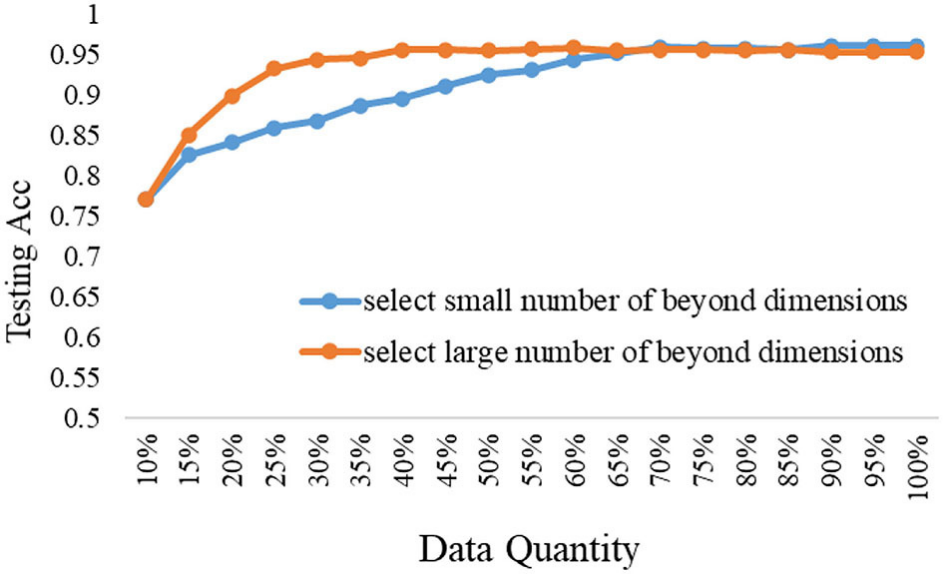
The data selection strategy of the ERJ method under the deep model.

The experimental results under the shallow model and the deep model are shown in Figures 6, 7, respectively.

It is shown from the above results that the data selection strategy of the ERJ method is closely related to the used model. If the used model is deep and has strong representational performance, then it should select the data with a large difference from the existing base data; that is, the number of beyond dimensions out of the existing embedding range is large. The main reason is that the deep model has enough ability to learn fast from the new unseen data. However, if the used model is shallow and has a weak representational performance, its overall testing performance is low, even if all data are used. In this case, it is better to select the data without too much difference from the existing base data at the first step, which is also supported by another theory called “self-paced learning.” The samples with a small number of beyond dimensions out of the existing embedding range mean that they are not too hard for the shallow model at this stage. Thus, the shallow model can incrementally improve performance. Then, after the first step, the differences between the two data selection strategies of the ERJ method are minor.

### Good Data vs. Bad Data Under Different Models

To further explore the trade-off of data quality and quantity, we carried out the comparison experiments to show the differences in testing performance based on good data or bad data under the three depth model. Notably, the good data refer to the selected data from the pool data that a large number of feature dimensions are out of the existing embedding range, while the bad data refer to the selected data from the pool data that all dimensions are inside the existing embedding range.

**Figure 8:**
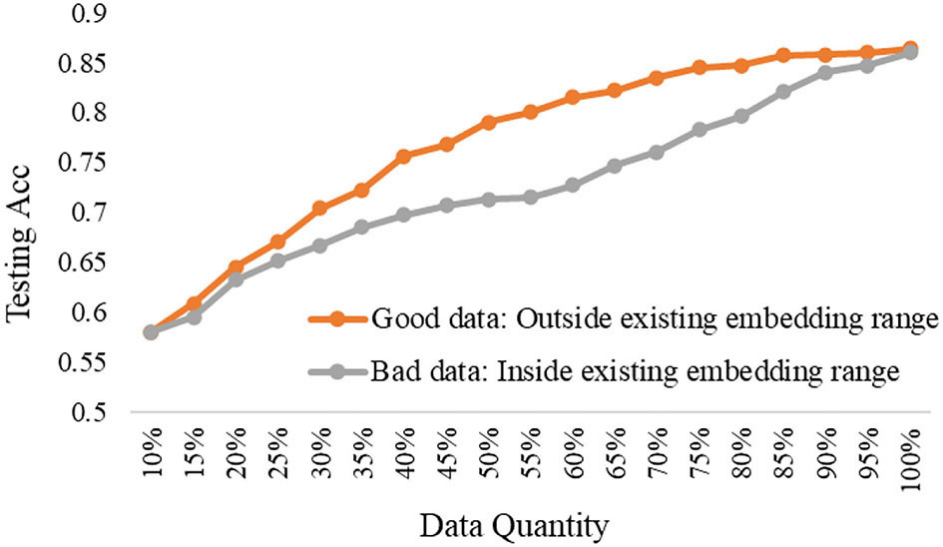
The good data vs. bad data under the shallow model.

**Figure 9:**
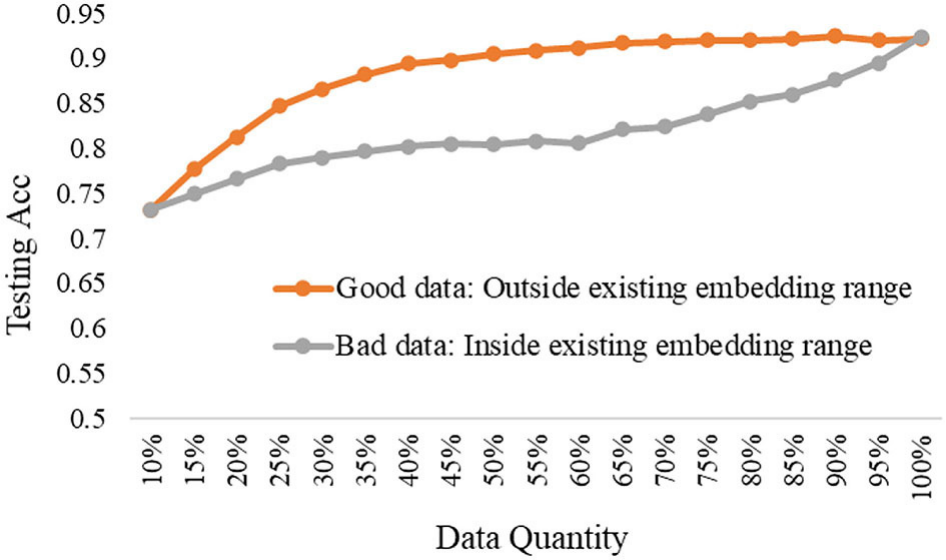
The good data vs. bad data under the middle model.

**Figure 10:**
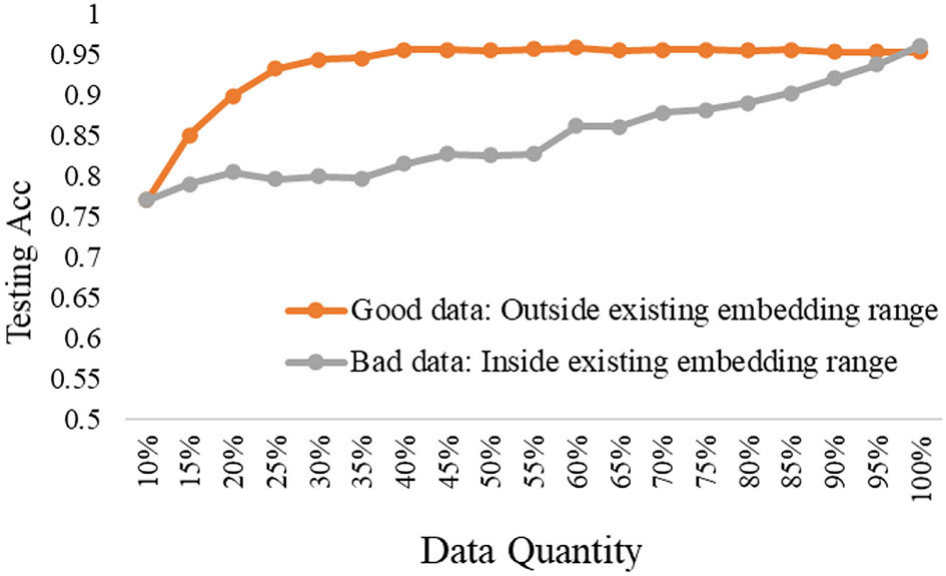
The good data vs. bad data under the deep model.

The experimental results are shown in Figures 8–10, according to the shallow model, middle model, and deep model, respectively.

The above results show the importance of the data quality analysis, and the comparison differences between good data and bad data are huge. The trends have been proved by different models according to the above curves. Specifically, we can conclude two basic findings in this section. The first is using the same data quantity, the model performance can be quite different based on good data or bad data, which can be distinguished by our proposed ERJ method. The other is aiming at the same model performance, the required data can be a small amount of high-quality good data or a large amount of poor-quality bad data. Moreover, this kind of difference will be more obvious when the used model has a strong representation learning ability.

## Discussions

In this section, we discuss this study from the following four aspects: motivation, contributions, reasons, and limitations and future work.

### Motivation

At present, the typical implementation of artificial intelligence (AI) technology on crop pest identification is deep learning, which, indeed has an effect when a large number of labeled data can be provided. But the necessity and sustainability are worth exploring. As mentioned, the existing big data cost, high-performance hardware cost, and excessive power consumption have indicated the unsustainability of the current deep learning approach. This fact motivates us that the sustainable learning method should be paid more attention in the future, which is based on limited high-quality data.

To explore the possibility of sustainable learning in intelligent agriculture or general pattern recognition, the first important thing is to seriously look at the data, which is the base of all the following tasks. Thus, we proposed an ERJ method to analyze and select samples with high information value to the existing data. The ERJ method focused on the feature space and considered whether new information can be brought.

### Contributions

The contributions of this study are three-fold:

First, we used the proposed ERJ analysis method to select high information value data and found that the small amount of good selected data can reach the same testing performance on all the training data. Moreover, this finding is verified by the models with different depths to show its robustness.

Second, we further explored the selection strategy of the proposed ERJ method and found that it is closely related to the used network for crop pest classification. For the deep model with strong representational ability, it prefers samples with a large number of dimensions out of the existing embedding range. For the shallow network, consider the initial few selection data, it prefers samples with a small number of dimensions out of the existing embedding range.

Finally, the proposed ERJ method can be used not only to select good data but also to select bad data, which is beneficial to reduce the redundancy of the existing dataset. With the same amount of data, the comparison differences between good data and bad data on crop pest identification performance are huge. Limited good data can beat lots of bad data.

### Reasons

There are sufficient reasons to explain the above core findings of this study. First, the embedding is the mapped data representation after dimensionality reduction in the feature space, which replaces the traditional image pixel analysis. Given the existing embedding range in the feature space, it represents the scope of information in the existing base data. Thus, the ERJ method can be used to select the outside valuable sample to supplement the base data. Second, the feature mapping depends on the representational ability of the used model, typically, the deep model has better performance than the shallow model, hence, the selection strategy of the ERJ method varies. But the comparisons between the out-of-range and in-range samples are consistent, which are called good data and bad data in this study. Last, the limited good data can beat lots of bad data as the redundant samples will not bring any substantial help on the recognition task but even reduce the training efficiency. Hence, the trade-off between data quantity and quality is reasonable and feasible for the computer vision tasks in smart agriculture.

### Limitations and Future Work

This study used random few data as initial data to fine-tune the model and get the feature embedding, which does not affect the reliability of the experimental results and trends, but there may be some limitations on the specific model performance analysis. The simple reason is that better model performance can be obtained based on good initial data. So, part of the future work will be put on the selection and analysis of the initial data. Besides, the sustainable learning methods not only consider the data aspect but also the model aspect. Thus, there will also be another part of work focused on the optimization of the lightweight network to better assist the analysis and selection of samples in the feature space.

## Conclusion

To reduce the data cost of crop pest identification and explore the trade-off between data quality and quantity, this study proposed an ERJ feature analysis method and carried out many comparative experiments. The results showed that there really is a combination of good data and less quantity, some small amount of good selected data can reach the same testing performance on all the training data. Specifically, the selection strategy of the proposed ERJ method depends on the representational ability of the used models. Furthermore, the limited good data can beat a lot of bad data, and the contrast is remarkable. Overall, this study lays a foundation for data information analysis in smart agriculture, inspires the promotion of subsequent related works, and calls for the community to pay more attention to the issues of data quality and quantity, aiming at changing the current unsustainable deep learning paradigm based on simply collecting large amounts of data.

## Data Availability Statement

The original contributions presented in the study are included in the article/supplementary materials, further inquiries can be directed to the corresponding author/s.

## Author Contributions

YL contributed to the conception, design of the study, and wrote the first draft of the manuscript. XC organized the database and performed the statistical analysis. Both authors contributed to manuscript revision, read, and approved the submitted version.

## Funding

This study was supported by the National Natural Science Foundation of China (No. 32101612) and the Major Science and Technology Projects of Xinjiang Production and Construction Corps (No. 2021AA006).

## Conflict of Interest

The authors declare that the research was conducted in the absence of any commercial or financial relationships that could be construed as a potential conflict of interest.

## Publisher's Note

All claims expressed in this article are solely those of the authors and do not necessarily represent those of their affiliated organizations, or those of the publisher, the editors and the reviewers. Any product that may be evaluated in this article, or claim that may be made by its manufacturer, is not guaranteed or endorsed by the publisher.

## References

[B1] Ayan E. Erbay H. Varçin F. (2020). Crop pest classification with a genetic algorithm-based weighted ensemble of deep convolutional neural networks. Comput. Electron. Agric. 179:105809. 10.1016/j.compag.2020.105809

[B2] Chao X. Zhang L. (2021). Few-shot imbalanced classification based on data augmentation. Multimedia Syst. 27, 1–9. 10.1007/s00530-021-00827-0

[B3] Chen Y. Liu S. Wu H. Zhang X. Zhou Q. (2020a). How can Belt and Road countries contribute to glocal low-carbon development? J. Clean. Product. 256:120717. 10.1016/j.jclepro.2020.120717

[B4] Chen Y. Zhang B. Zhou J. Wang K. (2020b). Real-time 3D unstructured environment reconstruction utilizing VR and Kinect-based immersive teleoperation for agricultural field robots. Comput. Electron. Agric. 175:105579. 10.1016/j.compag.2020.105579

[B5] Emmi L. Le Flécher E. Cadenat V. Devy M. (2021). A hybrid representation of the environment to improve autonomous navigation of mobile robots in agriculture. Precis. Agric. 22, 524–549. 10.1007/s11119-020-09773-9

[B6] Friha O. Ferrag M. A. Shu L. Maglaras L. A. Wang X. (2021). Internet of things for the future of smart agriculture: a comprehensive survey of emerging technologies. IEEE CAA J. Autom. Sin. 8, 718–752. 10.1109/JAS.2021.100392527295638

[B7] Fu L. Feng Y. Wu J. Liu Z. Gao F. Majeed Y.. (2021). Fast and accurate detection of kiwifruit in orchard using improved YOLOv3-tiny model. Precis. Agric. 22, 754–776. 10.1007/s11119-020-09754-y

[B8] Gao F. Fu L. Zhang X. Majeed Y. Li R. Karkee M.. (2020). Multi-class fruit-on-plant detection for apple in SNAP system using Faster R-CNN. Comput. Electron. Agric. 176:105634. 10.1016/j.compag.2020.105634

[B9] Guo N. Zhang B. Zhou J. Zhan K. Lai S. (2020). Pose estimation and adaptable grasp configuration with point cloud registration and geometry understanding for fruit grasp planning. Comput. Electron. Agric. 179:105818. 10.1016/j.compag.2020.105818

[B10] Jarlan L. Er-Raki S. Balaghi R. Amazirh A. Richard B. Khabba S. (2021). Cereal yield forecasting with satellite drought-based indices, weather data and regional climate indices using machine learning in Morocco. Remote Sens. 13:3101. 10.3390/rs13163101

[B11] Kanagasingham S. Ekpanyapong M. Chaihan R. (2020). Integrating machine vision-based row guidance with GPS and compass-based routing to achieve autonomous navigation for a rice field weeding robot. Precis. Agric. 21, 831–855. 10.1007/s11119-019-09697-z

[B12] Li J. Zhang L. Huang G. Wang H. Jiang Y. (2021). Experimental study on creep properties prediction of reed bales based on SVR and MLP. Plant Methods 17:112. 10.1186/s13007-021-00814-6 34717667 PMC8556900

[B13] Li Y. Chao X. (2020). ANN-based continual classification in agriculture. Agriculture 10:178. 10.3390/agriculture10050178

[B14] Li Y. Chao X. (2021). Semi-supervised few-shot learning approach for plant diseases recognition. Plant Methods 17:68. 10.1186/s13007-021-00770-1 34176505 PMC8237441

[B15] Li Y. Nie J. Chao X. (2020). Do we really need deep CNN for plant diseases identification? Comput. Electron. Agric. 178:105803. 10.1016/j.compag.2020.105803

[B16] Li Y. Yang J. (2020). Few-shot cotton pest recognition and terminal realization. Comput. Electron. Agric. 169:105240. 10.1016/j.compag.2020.105240

[B17] Li Y. Yang J. (2021). Meta-learning baselines and database for few-shot classification in agriculture. Comput. Electron. Agric. 182:106055. 10.1016/j.compag.2021.106055

[B18] Liang X. (2021). Few-shot cotton leaf spots disease classification based on metric learning. Plant Methods 17:114. 10.1186/s13007-021-00813-7 34749780 PMC8576888

[B19] Liu J. Wang X. (2020). Tomato diseases and pests detection based on improved Yolo V3 convolutional neural network. Front. Plant Sci. 11:898. 10.3389/fpls.2020.00898 32612632 PMC7309963

[B20] Mandal D. S. Chekroun A. Samanta S. Chattopadhyay J. (2021). A mathematical study of a crop-pest–natural enemy model with Z-type control. Math. Comput. Simul. 187, 468–488. 10.1016/j.matcom.2021.03.014

[B21] Nagasubramanian K. Jones S. Singh A. K. Sarkar S. Singh A. Ganapathysubramanian B. (2019). Plant disease identification using explainable 3D deep learning on hyperspectral images. Plant Methods 15:98. 10.1186/s13007-019-0479-8 31452674 PMC6702735

[B22] Pathan M. Patel N. Yagnik H. Shah M. (2020). Artificial cognition for applications in smart agriculture: a comprehensive review. Artif. Intell. Agric. 4, 81–95. 10.1016/j.aiia.2020.06.00134249054

[B23] Rovira-Más F. Saiz-Rubio V. Cuenca-Cuenca A. (2020). Augmented perception for agricultural robots navigation. IEEE Sens. J. 21, 11712–11727. 10.1109/JSEN.2020.301608127295638

[B24] Schauberger B. Jägermeyr J. Gornott C. (2020). A systematic review of local to regional yield forecasting approaches and frequently used data resources. Eur. J. Agron. 120:126153. 10.1016/j.eja.2020.126153

[B25] Shahhosseini M. Hu G. Archontoulis S. (2020). Forecasting corn yield with machine learning ensembles. Front. Plant Sci. 11:1120. 10.3389/fpls.2020.01120 32849688 PMC7411227

[B26] Wang R. Jiao L. Xie C. Chen P. Du J. Li R. (2021). S-RPN: sampling-balanced region proposal network for small crop pest detection. Comput. Electron. Agric. 187:106290. 10.1016/j.compag.2021.106290

[B27] Wen B. Q. Li Y. Kan Z. Li J. B. Li L. Ge J.. (2020). Experimental research on the bending characteristics of *Glycyrrhiza glabra* stems. Trans. ASABE 63, 1499–1506. 10.13031/trans.13802

[B28] Yang X. Shu L. Chen J. Ferrag M. A. Wu J. Nurellari E.. (2020). A survey on smart agriculture: development modes, technologies, and security and privacy challenges. IEEE/CAA J. Automat. Sin. 8, 273–302. 10.1109/JAS.2020.100353627295638

[B29] Yang Y. Zhang Z. Mao W. Li Y. Lv C. (2021). Radar target recognition based on few-shot learning. Multimedia Syst. 27, 1–11. 10.1007/s00530-021-00832-3

[B30] Zhang B. Xie Y. Zhou J. Wang K. Zhang Z. (2020). State-of-the-art robotic grippers, grasping and control strategies, as well as their applications in agricultural robots: a review. Comput. Electron. Agric. 177:105694. 10.1016/j.compag.2020.105694

